# The genome sequence of the Poplar Admiral, *Limenitis populi* (Linnaeus, 1758) (Lepidoptera: Nymphalidae)

**DOI:** 10.12688/wellcomeopenres.24725.2

**Published:** 2026-02-18

**Authors:** Jaakko Pohjoismaki, Marko Mutanen, Charlotte J. Wright, Joana I. Meier, Mark L. Blaxter, Wellcome Sanger Institute Tree of Life Management, Samples and Laboratory team, Wellcome Sanger Institute Scientific Operations: Sequencing Operations, Wellcome Sanger Institute Tree of Life Core Informatics team, Tree of Life Core Informatics collective, Project Psyche Community

**Affiliations:** 1 University of Eastern Finland, Joensuu, North Karelia, Finland; 2 University of Oulu, Oulu, Northern Ostrobothnia, Finland; 3 Tree of Life, Wellcome Sanger Institute, Hinxton, England, UK

**Keywords:** Limenitis populi, Poplar Admiral, genome sequence, chromosomal, Lepidoptera

## Abstract

We present a genome assembly from a male specimen of *Limenitis populi* (Poplar Admiral; Arthropoda; Insecta; Lepidoptera; Nymphalidae). The assembly contains two haplotypes with total lengths of 342.08 megabases and 341.73 megabases. Most of haplotype 1 (99.97%) is scaffolded into 30 chromosomal pseudomolecules, including the Z sex chromosome. Haplotype 2 was assembled to scaffold level. The mitochondrial genome has also been assembled, with a length of 15.22 kilobases. Gene annotation of this assembly on Ensembl identified 11 495 protein-coding genes. This work is part of Project Psyche, a collaborative programme generating genomes for European butterflies and moths.

## Species taxonomy

Eukaryota; Opisthokonta; Metazoa; Eumetazoa; Bilateria; Protostomia; Ecdysozoa; Panarthropoda; Arthropoda; Mandibulata; Pancrustacea; Hexapoda; Insecta; Dicondylia; Pterygota; Neoptera; Endopterygota; Amphiesmenoptera; Lepidoptera; Glossata; Neolepidoptera; Heteroneura; Ditrysia; Obtectomera; Papilionoidea; Nymphalidae; Limenitidinae; Limenitidini; *Limenitis*; *Limenitis populi* (Linnaeus, 1758) (NCBI:txid227535).

## Background

The Poplar Admiral ( *Limenitis populi*) is a striking butterfly in the family Nymphalidae. Adults are large, with a wingspan of 70–90 mm, and are easily identified by their dark brown to black wings adorned with a prominent white band and orange spots on the hindwings. The underside is equally distinctive, with intricate orange and white markings. The species is found across much of Europe and Asia, favouring deciduous and mixed woodlands with abundant aspen ( *Populus tremula*), its primary larval host plant ( Silvonen *et al*., 2014).

This species is univoltine, with adults on wing between late June and early August, females starting their flight earlier than the males. Poplar Admirals are often difficult to observe as they rarely descend from the canopy and almost never visit flowers. Instead, they are often found feeding on faeces, urine or carrion. Females lay eggs on aspen leaves, rarely other poplar species. The larvae hatch in late summer and feed on the leaves before overwintering as small caterpillars in a hibernaculum. Development resumes in spring, with pupation occurring in early summer.

Although *L. populi* is widespread, its populations in mainland Europe are often patchy and sensitive to habitat fragmentation. For instance, the species is listed as critically endangered (CR) in Germany ( Reinhardt & Bolz, 2011). In contrast, populations in the northern parts of the species’ distribution have witnessed an impressive climate change-driven range expansion over the last couple of decades ( Saarinen & Jantunen, 2013; Sunde *et al*., 2023).

**Figure 1.**
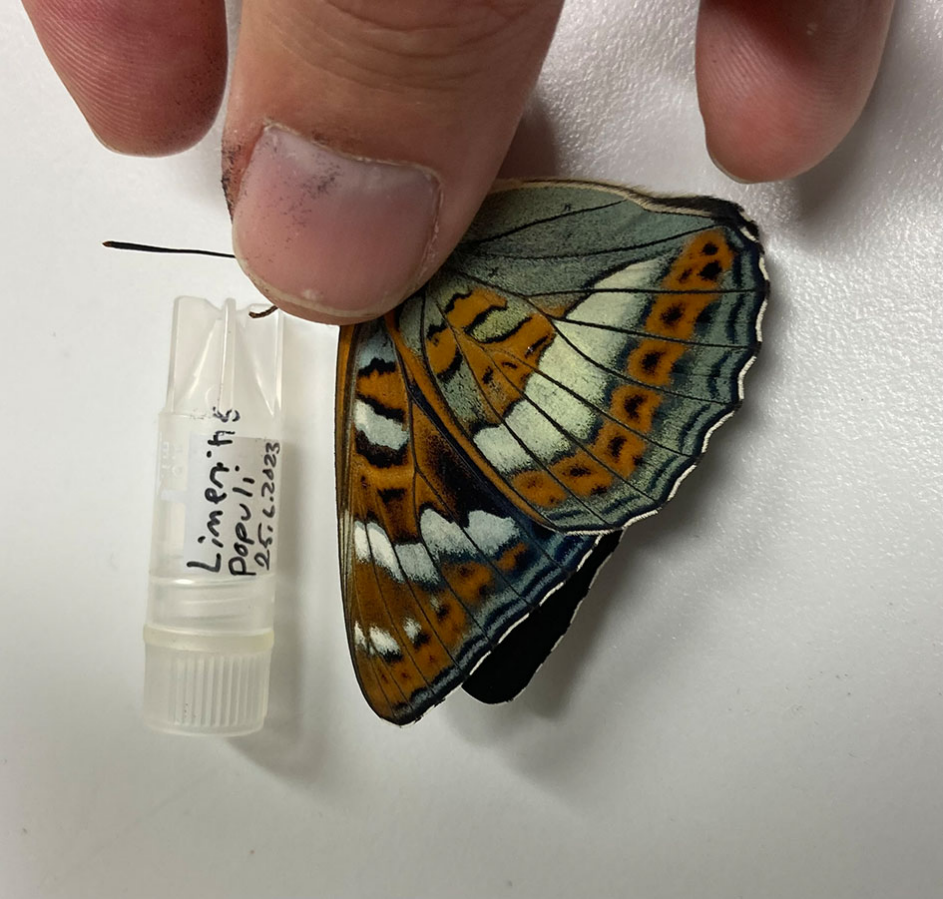
Photograph of the *Limenitis populi* (ilLimPopu1) specimen used for genome sequencing.

The reference genome assembly for *Limenitis populi* presented here is a valuable addition to the expanding genomic resources for Nymphalid butterflies. It provides a foundation for investigating the genetic basis of the species’ ecological adaptations, including host plant specialisation and overwintering strategies. Additionally, this genome will support comparative studies within the genus *Limenitis*, the subfamily Heliconiinae and the broader family Nymphalidae, which include model taxa extensively studied for wing pattern evolution ( Joron *et al*., 2006). The sequence data were derived from a male specimen ( Figure 1) collected from Hautalahti, Liperi, North Karelia, Finland.

## Methods

### Sample acquisition

The specimen used for genome sequencing was an adult male *Limenitis populi* (specimen ID SAN28000003, ToLID ilLimPopu1; Figure 1), collected from Hautalahti, Liperi, North Karelia, Finland (latitude 62.5561, longitude 29.1041). The specimen was collected and identified by Jaakko Pohjoismaki (University of Eastern Finland).

### Nucleic acid extraction

Protocols for high molecular weight (HMW) DNA extraction developed at the Wellcome Sanger Institute (WSI) Tree of Life Core Laboratory are available on protocols.io ( Howard *et al*., 2025). The ilLimPopu1 sample was weighed and triaged to determine the appropriate extraction protocol. Tissue from the whole organism was homogenised by powermashing using a PowerMasher II tissue disruptor.

HMW DNA was extracted in the WSI Scientific Operations core using the Automated MagAttract v2 protocol. DNA was sheared into an average fragment size of 12–20 kb following the Megaruptor®3 for LI PacBio protocol. Sheared DNA was purified by automated SPRI (solid-phase reversible immobilisation). The concentration of the sheared and purified DNA was assessed using a Nanodrop spectrophotometer and Qubit Fluorometer using the Qubit dsDNA High Sensitivity Assay kit. Fragment size distribution was evaluated by running the sample on the FemtoPulse system. For this sample, the final post-shearing DNA had a Qubit concentration of 29.13 ng/μL and a yield of 1 369.11 ng, with a fragment size of 17.9 kb.

RNA was extracted from whole organism tissue of ilLimPopu1 in the Tree of Life Laboratory at the WSI using the RNA Extraction: Automated MagMax™ *mir*Vana protocol. The RNA concentration was assessed using a Nanodrop spectrophotometer and a Qubit Fluorometer using the Qubit RNA Broad-Range Assay kit. Analysis of the integrity of the RNA was done using the Agilent RNA 6000 Pico Kit and Eukaryotic Total RNA assay.

### PacBio HiFi library preparation and sequencing

Library preparation and sequencing were performed at the WSI Scientific Operations core. Libraries were prepared using the SMRTbell Prep Kit 3.0 (Pacific Biosciences, California, USA), following the manufacturer’s instructions. The kit includes reagents for end repair/A-tailing, adapter ligation, post-ligation SMRTbell bead clean-up, and nuclease treatment. Size selection and clean-up were performed using diluted AMPure PB beads (Pacific Biosciences). DNA concentration was quantified using a Qubit Fluorometer v4.0 (ThermoFisher Scientific) and the Qubit 1X dsDNA HS assay kit. Final library fragment size was assessed with the Agilent Femto Pulse Automated Pulsed Field CE Instrument (Agilent Technologies) using the gDNA 55 kb BAC analysis kit.

The sample was sequenced on a Revio instrument (Pacific Biosciences). The prepared library was normalised to 2 nM, and 15 μL was used for making complexes. Primers were annealed and polymerases bound to generate circularised complexes, following the manufacturer’s instructions. Complexes were purified using 1.2X SMRTbell beads, then diluted to the Revio loading concentration (200–300 pM) and spiked with a Revio sequencing internal control. The sample was sequenced on a Revio 25M SMRT cell. The SMRT Link software (Pacific Biosciences), a web-based workflow manager, was used to configure and monitor the run and to carry out primary and secondary data analysis.

Specimen details, sequencing platforms, and data yields are summarised in Table 1.

**Table 1.** Specimen and sequencing data for BioProject.

Platform	PacBio HiFi	Hi-C	RNA-seq
**ToLID**	ilLimPopu1	ilLimPopu1	ilLimPopu1
**Specimen ID**	SAN28000003	SAN28000003	SAN28000003
**BioSample (source individual)**	SAMEA114539582	SAMEA114539582	SAMEA114539582
**BioSample (tissue)**	SAMEA114539616	SAMEA114539616	SAMEA114539616
**Tissue**	whole organism	whole organism	whole organism
**Sequencing platform and model**	Revio	Illumina NovaSeq X	Illumina NovaSeq X
**Run accessions**	ERR13900449	ERR13907227	ERR14986718
**Read count total**	1.63 million	840.62 million	154.66 million
**Base count total**	17.93 Gb	126.93 Gb	23.35 Gb

### Hi-C

***Sample preparation and crosslinking***

The Hi-C sample was prepared from 20–50 mg of frozen whole organism tissue of the ilLimPopu1 sample using the Arima-HiC v2 kit (Arima Genomics). Following the manufacturer’s instructions, tissue was fixed and DNA crosslinked using TC buffer to a final formaldehyde concentration of 2%. The tissue was homogenised using the Diagnocine Power Masher-II. Crosslinked DNA was digested with a restriction enzyme master mix, biotinylated, and ligated. Clean-up was performed with SPRISelect beads before library preparation. DNA concentration was measured with the Qubit Fluorometer (Thermo Fisher Scientific) and Qubit HS Assay Kit. The biotinylation percentage was estimated using the Arima-HiC v2 QC beads.

***Hi-C library preparation and sequencing***

Biotinylated DNA constructs were fragmented using a Covaris E220 sonicator and size selected to 400–600 bp using SPRISelect beads. DNA was enriched with Arima-HiC v2 kit Enrichment beads. End repair, A-tailing, and adapter ligation were carried out with the NEBNext Ultra II DNA Library Prep Kit (New England Biolabs), following a modified protocol where library preparation occurs while DNA remains bound to the Enrichment beads. Library amplification was performed using KAPA HiFi HotStart mix and a custom Unique Dual Index (UDI) barcode set (Integrated DNA Technologies). Depending on sample concentration and biotinylation percentage determined at the crosslinking stage, libraries were amplified with 10–16 PCR cycles. Post-PCR clean-up was performed with SPRISelect beads. Libraries were quantified using the AccuClear Ultra High Sensitivity dsDNA Standards Assay Kit (Biotium) and a FLUOstar Omega plate reader (BMG Labtech).

Prior to sequencing, libraries were normalised to 10 ng/μL. Normalised libraries were quantified again and equimolar and/or weighted 2.8 nM pools. Pool concentrations were checked using the Agilent 4200 TapeStation (Agilent) with High Sensitivity D500 reagents before sequencing. Sequencing was performed using paired-end 150 bp reads on the Illumina NovaSeq X.

Specimen details, sequencing platforms, and data yields are summarised in Table 1.

### RNA-seq library preparation and sequencing

Libraries were prepared using the NEBNext ® Ultra™ II Directional RNA Library Prep Kit for Illumina (New England Biolabs), following the manufacturer’s instructions. Poly(A) mRNA in the total RNA solution was isolated using oligo (dT) beads, converted to cDNA, and uniquely indexed; 14 PCR cycles were performed. Libraries were size-selected to produce fragments between 100–300 bp. Libraries were quantified, normalised, pooled to a final concentration of 2.8 nM, and diluted to 150 pM for loading. Sequencing was carried out on the Illumina NovaSeq X to generate 150-bp paired-end reads.

### Genome assembly

Prior to assembly of the PacBio HiFi reads, a database of *k*-mer counts ( *k* = 31) was generated from the filtered reads using FastK. GenomeScope2 ( Ranallo-Benavidez *et al*., 2020) was used to analyse the *k*-mer frequency distributions, providing estimates of genome size, heterozygosity, and repeat content.

The HiFi reads were assembled using Hifiasm in Hi-C phasing mode ( Cheng *et al*., 2021; Cheng *et al*., 2022), producing two haplotypes. Hi-C reads ( Rao *et al*., 2014) were mapped to the primary contigs using bwa-mem2 ( Vasimuddin *et al*., 2019). Contigs were further scaffolded with Hi-C data in YaHS ( Zhou *et al*., 2023), using the --break option for handling potential misassemblies. The scaffolded assemblies were evaluated using Gfastats ( Formenti *et al*., 2022), BUSCO ( Manni *et al*., 2021) and Merqury.FK ( Rhie *et al*., 2020).

The mitochondrial genome was assembled using MitoHiFi ( Uliano-Silva *et al*., 2023), which runs MitoFinder ( Allio *et al*., 2020) and uses these annotations to select the final mitochondrial contig and to ensure the general quality of the sequence.

### Assembly curation

The assembly was decontaminated using the Assembly Screen for Cobionts and Contaminants ( ASCC) pipeline. TreeVal was used to generate the flat files and maps for use in curation. Manual curation was conducted primarily in PretextView and HiGlass ( Kerpedjiev *et al*., 2018). Scaffolds were visually inspected and corrected as described by Howe *et al*. (2021). Manual corrections included 11 breaks, 30 joins, and removal of 8 haplotypic duplications. The curation process is documented at https://gitlab.com/wtsi-grit/rapid-curation . PretextSnapshot was used to generate a Hi-C contact map of the final assembly.

### Assembly quality assessment

Chromosomal painting was performed using lep_busco_painter using Merian elements, which represent the 32 ancestral linkage groups in Lepidoptera ( Wright *et al*., 2024). Painting was based on gene locations from the lepidoptera_odb10 BUSCO analysis and chromosome lengths from the genome index produced using SAMtools faidx ( Danecek *et al*., 2021). Each complete BUSCO (including both single-copy and duplicated BUSCOs) was assigned to a Merian element using a reference database, and coloured positions were plotted along chromosomes drawn to scale.

The Merqury.FK tool ( Rhie *et al*., 2020), run in a Singularity container ( Kurtzer *et al*., 2017), was used to evaluate *k*-mer completeness and assembly quality for both haplotypes using the *k*-mer database ( *k* = 31) computed prior to genome assembly. The analysis outputs included assembly QV scores and completeness statistics.

The genome was analysed using the BlobToolKit pipeline, a Nextflow implementation of the earlier Snakemake BlobToolKit pipeline ( Challis *et al*., 2020). The pipeline aligns PacBio reads using minimap2 ( Li, 2018) and SAMtools ( Danecek *et al*., 2021) to generate coverage tracks. Simultaneously, it queries the GoaT database ( Challis *et al*., 2023) to identify relevant BUSCO lineages and runs BUSCO ( Manni *et al*., 2021). For the three domain-level BUSCO lineages, BUSCO genes are aligned to the UniProt Reference Proteomes database ( Bateman *et al*., 2023) using DIAMOND blastp (Buchfink *et al*., 2021). The genome is divided into chunks based on the density of BUSCO genes from the closest taxonomic lineage, and each chunk is aligned to the UniProt Reference Proteomes database with DIAMOND blastx. Sequences without hits are chunked using seqtk and aligned to the NT database with blastn ( Altschul *et al*., 1990). The BlobToolKit suite consolidates all outputs into a blobdir for visualisation. The BlobToolKit pipeline was developed using nf-core tooling ( Ewels *et al*., 2020) and MultiQC ( Ewels *et al*., 2016), with package management via Conda and Bioconda ( Grüning *et al*., 2018), and containerisation through Docker ( Merkel, 2014) and Singularity ( Kurtzer *et al*., 2017).

### Genome sequence report

***Sequence data***

The genome of a specimen of *Limenitis populi* was sequenced using Pacific Biosciences single-molecule HiFi long reads, generating 17.93 Gb (gigabases) from 1.63 million reads, which were used to assemble the genome. GenomeScope2.0 analysis estimated the haploid genome size at 348.54 Mb, with a heterozygosity of 0.16% and repeat content of 13.97%. These estimates guided expectations for the assembly. Based on the estimated genome size, the sequencing data provided approximately 50× coverage. Hi-C sequencing produced 126.93 Gb from 840.62 million reads, which were used to scaffold the assembly. RNA sequencing data were also generated and are available in public sequence repositories. Table 1 summarises the specimen and sequencing details.

### Assembly statistics

The genome was assembled into two haplotypes using Hi-C phasing. Haplotype 1 was curated to chromosome level, while haplotype 2 was assembled to scaffold level. The final assembly has a total length of 342.08 Mb in 33 scaffolds, with 80 gaps, and a scaffold N50 of 12.4 Mb ( Table 2).

**Table 2.** Genome assembly statistics.

Assembly name	ilLimPopu1.hap1.1	ilLimPopu1.hap2.1
**Assembly accession**	GCA_964341245.1	GCA_964341235.1
**Assembly level**	chromosome	scaffold
**Span (Mb)**	342.08	341.73
**Number of chromosomes**	30	scaffold-level
**Number of contigs**	113	115
**Contig N50**	6.29 Mb	6.17 Mb
**Number of scaffolds**	33	53
**Scaffold N50**	12.4 Mb	12.37 Mb
**Longest scaffold length (Mb)**	21.97	-
**Sex chromosomes**	Z	-
**Organelles**	Mitochondrial genome: 15.22 kb	-

**Figure 2.**
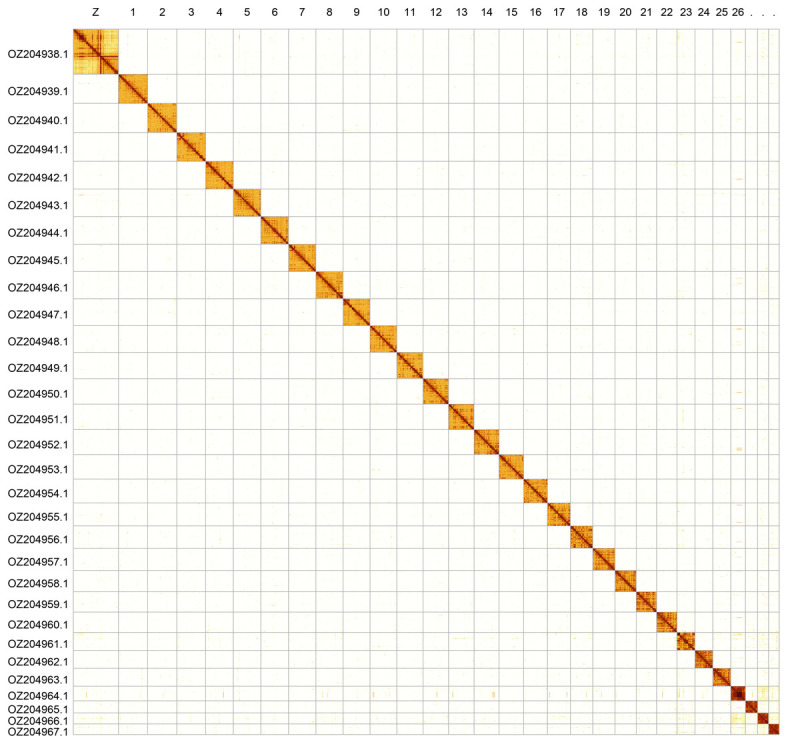
Hi-C contact map of the *Limenitis populi* genome assembly. Assembled chromosomes are shown in order of size and labelled along the axes. The plot was generated using PretextSnapshot.

**Figure 3.**
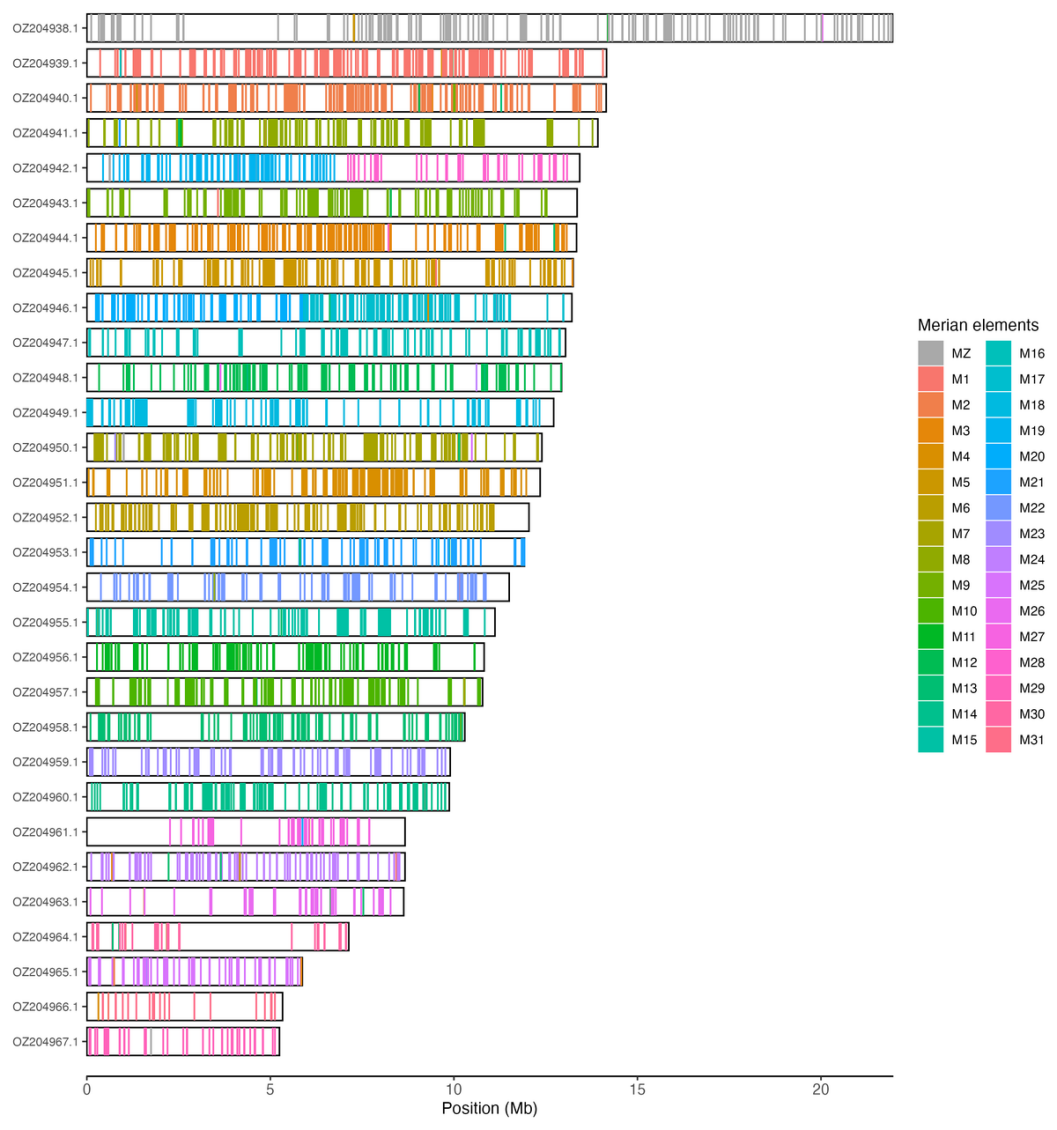
Merian elements painted across chromosomes in the ilLimPopu1.hap1.1 assembly of *Limenitis populi.* Chromosomes are drawn to scale, with the positions of orthologues shown as coloured bars. Each orthologue is coloured by the Merian element that it belongs to. All orthologues which could be assigned to Merian elements are shown.

Most of the assembly sequence (99.97%) was assigned to 30 chromosomal-level scaffolds, representing 29 autosomes and the Z sex chromosome. Chromosome Z was identified by alignment to the genome of *Limenitis camilla* (GCA_905147385.1) ( Vila *et al*., 2022). Scaffolds have uncertain order and orientation on Chromosome 23 in the approximate region 0.41–1.19 Mb, and Chromosome 26 in the region 2.98–5.37 Mb. The chromosome-level scaffolds, confirmed by Hi-C data, are named according to size ( Figure 2; Table 3). Chromosome painting with Merian elements illustrates the distribution of orthologues along chromosomes and highlights patterns of chromosomal evolution relative to Lepidopteran ancestral linkage groups ( Figure 3).

**Table 3.** Chromosomal pseudomolecules in the haplotype 1 genome assembly of *Limenitis populi* ilLimPopu1.

INSDC accession	Molecule	Length (Mb)	GC%	Assigned Merian elements
OZ204939.1	1	14.16	32.50	M1
OZ204940.1	2	14.15	32.50	M2
OZ204941.1	3	13.92	32.50	M8
OZ204942.1	4	13.43	32	M19;M28
OZ204943.1	5	13.36	32	M9
OZ204944.1	6	13.34	32.50	M3
OZ204945.1	7	13.25	33	M5
OZ204946.1	8	13.21	32.50	M17;M20
OZ204947.1	9	13.04	32.50	M16
OZ204948.1	10	12.93	32.50	M12
OZ204949.1	11	12.72	32	M18
OZ204950.1	12	12.40	32.50	M7
OZ204951.1	13	12.35	32.50	M4
OZ204952.1	14	12.04	32.50	M6
OZ204953.1	15	11.92	32	M21
OZ204954.1	16	11.51	32.50	M22
OZ204955.1	17	11.12	32.50	M15
OZ204956.1	18	10.82	33	M11
OZ204957.1	19	10.78	32.50	M10
OZ204958.1	20	10.30	32.50	M13
OZ204959.1	21	9.90	33	M23
OZ204960.1	22	9.87	33	M14
OZ204961.1	23	8.67	35.50	M27
OZ204962.1	24	8.67	33	M24
OZ204963.1	25	8.63	33	M26
OZ204964.1	26	7.14	38	M30
OZ204965.1	27	5.87	33.50	M25
OZ204966.1	28	5.33	35	M31
OZ204967.1	29	5.25	34	M29
OZ204938.1	Z	21.97	32.50	MZ

The mitochondrial genome was also assembled (length 15.22 kb, OZ204968.1). This sequence is included as a contig in the multifasta file of the genome submission and as a standalone record.

### Assembly quality metrics

**Figure 4.**
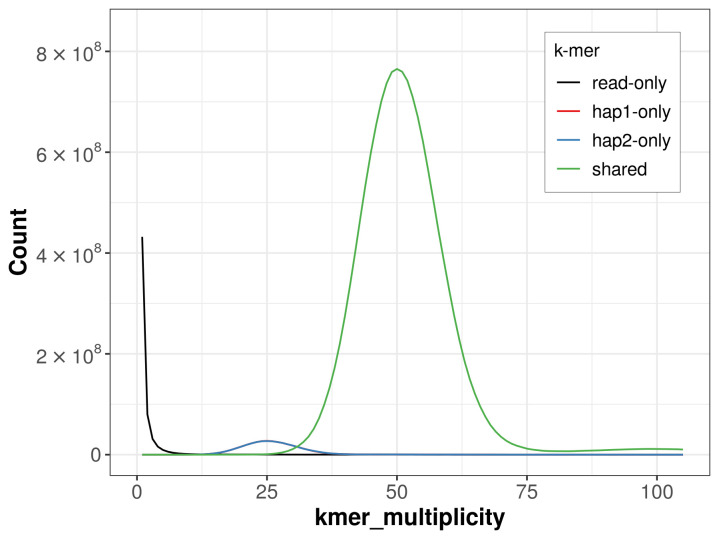
Evaluation of *k*-mer completeness using MerquryFK. This plot illustrates the recovery of *k*-mers from the original read data in the final assemblies. The horizontal axis represents *k*-mer multiplicity, and the vertical axis shows the number of *k*-mers. The black curve represents *k*-mers that appear in the reads but are not assembled. The green curve (the homozygous peak) corresponds to *k*-mers shared by both haplotypes and the red and blue curves (the heterozygous peaks) show *k*-mers found only in one of the haplotypes.

**Figure 5.**
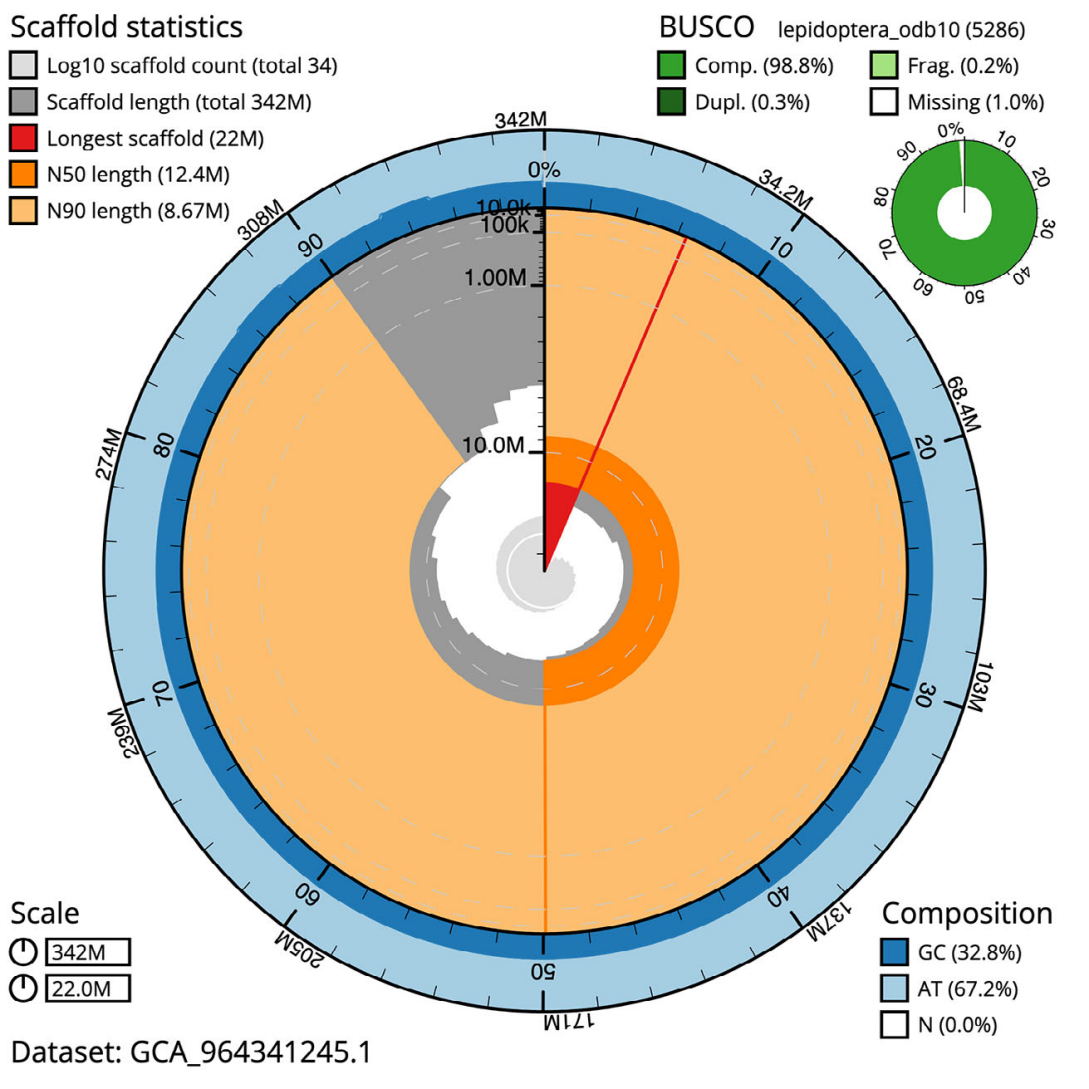
Assembly metrics for ilLimPopu1.hap1.1. The BlobToolKit snail plot provides an overview of assembly metrics and BUSCO gene completeness. The circumference represents the length of the whole genome sequence, and the main plot is divided into 1,000 bins around the circumference. The outermost blue tracks display the distribution of GC, AT, and N percentages across the bins. Scaffolds are arranged clockwise from longest to shortest and are depicted in dark grey. The longest scaffold is indicated by the red arc, and the deeper orange and pale orange arcs represent the N50 and N90 lengths. A light grey spiral at the centre shows the cumulative scaffold count on a logarithmic scale. A summary of complete, fragmented, duplicated, and missing BUSCO genes in the set is presented at the top right. An interactive version of this figure can be accessed on the BlobToolKit viewer.

**Figure 6.**
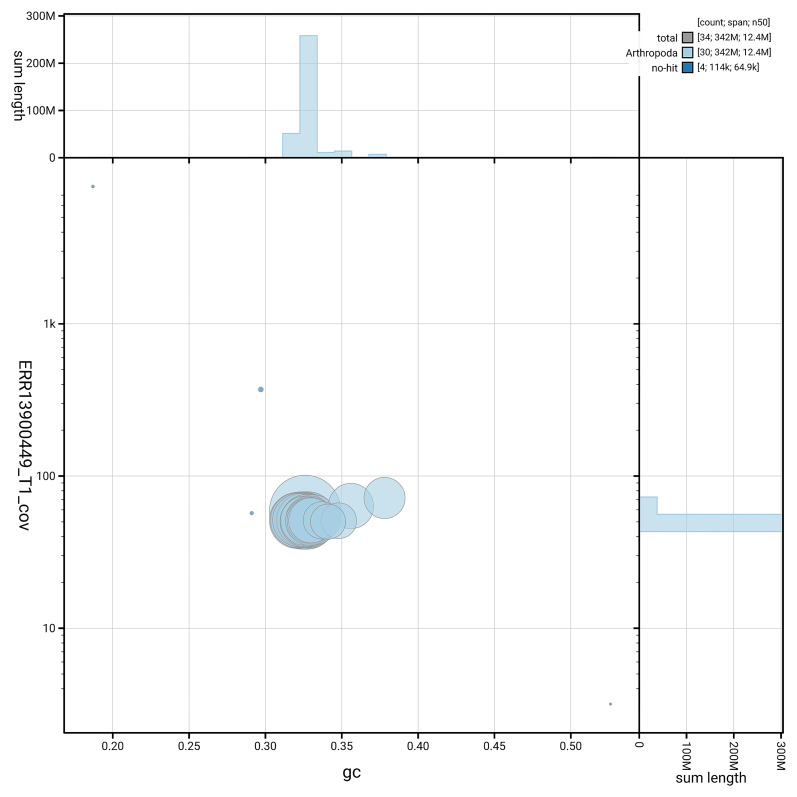
BlobToolKit GC-coverage plot for ilLimPopu1.hap1.1. Blob plot showing sequence coverage (vertical axis) and GC content (horizontal axis). The circles represent scaffolds, with the size proportional to scaffold length and the colour representing phylum membership. The histograms along the axes display the total length of sequences distributed across different levels of coverage and GC content. An interactive version of this figure is available on the BlobToolKit viewer.

For haplotype 1, the estimated QV is 61.7, and for haplotype 2, 62.7. When the two haplotypes are combined, the assembly achieves an estimated QV of 62.1. The *k*-mer completeness is 95.05% for haplotype 1, 95.06% for haplotype 2, and 99.46% for the combined haplotypes ( Figure 4). BUSCO analysis using the lepidoptera_odb10 reference set ( *n* = 5 286) ( Kriventseva *et al*., 2019) identified 98.8% of the expected gene set (single = 98.5%, duplicated = 0.3%) for haplotype 1. The snail plot in Figure 5 summarises the scaffold length distribution and other assembly statistics for haplotype 1. The blob plot in Figure 6 shows the distribution of scaffolds by GC proportion and coverage for haplotype 1.

Table 4 lists the assembly metric benchmarks adapted from Rhie *et al.* (2021) the Earth BioGenome Project Report on Assembly Standards September 2024. The EBP metric, calculated for the haplotype 1, is **6.C.Q61**, meeting the recommended reference standard.

**Table 4.** Earth Biogenome Project summary metrics for the *Limenitis populi* assembly.

Measure	Value	Benchmark
EBP summary (haplotype 1)	6.7.Q61	6.C.Q40
Contig N50 length	6.29 Mb	≥ 1 Mb
Scaffold N50 length	12.40 Mb	= chromosome N50
Consensus quality (QV)	Haplotype 1: 61.7; haplotype 2: 62.7; combined: 62.1	≥ 40
*k*-mer completeness	Haplotype 1: 95.05%; Haplotype 2: 95.06%; combined: 99.46%	≥ 95%
BUSCO	C:98.8%[S:98.5%‚D:0.3%]‚ F:0.2%‚M:1.0%‚n:5 286	S > 90%; D < 5%
Percentage of assembly assigned to chromosomes	99.97%	≥ 90%

### Genome annotation report

The *Limenitis populi* genome assembly (GCA_964341245.1) was annotated by Ensembl at the European Bioinformatics Institute (EBI). This annotation includes 20 563 transcribed mRNAs from 11 495 protein-coding and 1 654 non-coding genes. The average transcript length is 13 302.72 bp, with an average of 1.56 coding transcripts per gene and 7.92 exons per transcript. For further information, please refer to the Ensembl annotation page.

### Wellcome Sanger Institute – Legal and Governance

The materials that have contributed to this genome note have been supplied by a Tree of Life collaborator. The Wellcome Sanger Institute employs a process whereby due diligence is carried out proportionate to the nature of the materials themselves, and the circumstances under which they have been/are to be collected and provided for use. The purpose of this is to address and mitigate any potential legal and/or ethical implications of receipt and use of the materials as part of the research project, and to ensure that in doing so, we align with best practice wherever possible. The overarching areas of consideration are:

• Ethical review of provenance and sourcing of the material
• Legality of collection, transfer and use (national and international).

Each transfer of samples is undertaken according to a Research Collaboration Agreement or Material Transfer Agreement entered into by the Tree of Life collaborator, Genome Research Limited (operating as the Wellcome Sanger Institute), and in some circumstances, other Tree of Life collaborators.

## Data Availability

European Nucleotide Archive: Limenitis populi (poplar admiral). Accession number PRJEB81614. The genome sequence is released openly for reuse. The *Limenitis populi* genome sequencing initiative is part of the Sanger Institute Tree of Life Programme (PRJEB43745) and Project Psyche (PRJEB71705). All raw sequence data and the assembly have been deposited in INSDC databases. The genome will be annotated using available RNA-Seq data and presented through Ensembl at the European Bioinformatics Institute. Raw data and assembly accession identifiers are reported in Table 1 and Table 2. Pipelines used for genome assembly at the WSI Tree of Life are available at https://pipelines.tol.sanger.ac.uk/pipelines. Table 5 lists software versions used in this study.

## Author information

Contributors are listed at the following links:

• Wellcome Sanger Institute Tree of Life Management, Samples and Laboratory team
• Wellcome Sanger Institute Scientific Operations – Sequencing Operations
• Wellcome Sanger Institute Tree of Life Core Informatics team
• Tree of Life Core Informatics collective
• Project Psyche Community.
