# Supplementary material for: The genome sequence of the Poplar Admiral, Limenitis populi (Linnaeus, 1758) (Lepidoptera: Nymphalidae)
Source: Wellcome Open Res. 2026 Feb 18;10:438. Originally published 2025 Aug 11. [Version 2] doi: 10.12688/wellcomeopenres.24725.2 (PMC12921399; doi:10.12688/wellcomeopenres.24725.2)
Supplement: Peer review history

# Reviewer report: Reviewer response for version 2

**DOI:** 10.21956/wellcomeopenres.28662.r142690

**Published:** 2026-03-05

**Authors:** Annabel Whibley

**Affiliations:** 1 Grapevine Improvement, Bragato Research Institute, Lincoln, New Zealand

Pohjoismaki and colleagues report the genome assembly and annotation of the Poplar Admiral *(Limenitis populi),* performed under the auspices of Project Psyche *.  *I am reviewing version 2 of this report, which has amended two figures and newly linked to the Ensembl gene annotation. The natural history section is well written, and the report uses Darwin Tree of Life pipelines and templating- resulting in comprehensive reporting of metadata associated with the sample and bioinformatics processing. The specimen sequenced was male (hence no W chromosome) and species ID was not confirmed by barcoding.

 Both haplotypes were recovered in full, with haplotype 1 scaffolded into pseudochromosomes. Regions with uncertain order/orientation of scaffolds are noted. Assembly quality metrics are excellent and formally reach  6.C.Q61. Please note that Table 4 contains a templated error whereby the EBP summary value is incorrectly reported as 6.7.Q61.

Are sufficient details of methods and materials provided to allow replication by others?

Yes

Is the rationale for creating the dataset(s) clearly described?

Yes

Are the datasets clearly presented in a useable and accessible format?

Yes

Are the protocols appropriate and is the work technically sound?

Yes

Reviewer Expertise:

Genomics, Bioinformatics

I confirm that I have read this submission and believe that I have an appropriate level of expertise to confirm that it is of an acceptable scientific standard.

# Reviewer report: Reviewer response for version 2

**DOI:** 10.21956/wellcomeopenres.28662.r148182

**Published:** 2026-02-19

**Authors:** Sina Beier

**Affiliations:** 1 Gurdon Institute, Cambridge, England, UK

na

Are sufficient details of methods and materials provided to allow replication by others?

Yes

Is the rationale for creating the dataset(s) clearly described?

Yes

Are the datasets clearly presented in a useable and accessible format?

Yes

Are the protocols appropriate and is the work technically sound?

Yes

Reviewer Expertise:

bioinformatics

I confirm that I have read this submission and believe that I have an appropriate level of expertise to confirm that it is of an acceptable scientific standard.

# Reviewer report: Reviewer response for version 2

**DOI:** 10.21956/wellcomeopenres.28662.r148181

**Published:** 2026-02-19

**Authors:** Jose Ramon Pardos-Blas

**Affiliations:** 1 Real Jardin Botanico (Ringgold ID: 54423), Madrid, Community of Madrid, Spain

The authors have addressed my previous suggestions appropriately.

Are sufficient details of methods and materials provided to allow replication by others?

Yes

Is the rationale for creating the dataset(s) clearly described?

Yes

Are the datasets clearly presented in a useable and accessible format?

No

Are the protocols appropriate and is the work technically sound?

Yes

Reviewer Expertise:

genomics

I confirm that I have read this submission and believe that I have an appropriate level of expertise to confirm that it is of an acceptable scientific standard.

# Reviewer report: Reviewer response for version 1

**DOI:** 10.21956/wellcomeopenres.27242.r142687

**Published:** 2026-01-09

**Authors:** Jose Ramon Pardos-Blas

**Affiliations:** 1 Real Jardin Botanico (Ringgold ID: 54423), Madrid, Community of Madrid, Spain

The authors present the assembly of the lepidopteran *Limenitis populi*. The new genome is based on PacBio HiFi and Hi-C data, which meets state-of-the-art standards for high-quality assemblies. I found the introduction useful and informative about the species’ biology. The quality of the assembly is very good considering the metrics presented in Table 4. Since I do not have much experience with library preparation methods, I may not be able to fully evaluate these parts, but they appear to be well presented and provide access to the protocols used.

 My main critique is that, although RNA-seq was performed, no annotation has been carried out. This decreases the usefulness of the data as a community resource. I would like to suggest that the authors perform a genome annotation and include it in this report.

 Other comments to the authors:

 - In Figure 1, panel A is described as a “live specimen”, but it appears to be a museum specimen. Please clarify whether this represents a different specimen.

 - According to the text and Table 1, the material for the long reads, Hi-C, and RNA comes from the same specimen and the same bulk tissue. I would like to ask whether the method involved grinding the full body (excluding the wings) and splitting the tissue for the different extractions/libraries, or whether a different approach was followed. I recommend clarifying this in the text, as I found it confusing.

 - It is stated that “prior to assembly of the PacBio HiFi reads, a database of k-mer counts (k = 31) was generated from the filtered reads”; however, I was not able to find in the text how the reads were filtered. I recommend completing the Methods section with this information, together with the corresponding software versions in Table 5.

 - The Hi-C reads were mapped to the genome using bwa-mem2. While this is a valid approach, there are pipelines that implement additional steps to correct mapping errors and are commonly used, such as the Arima mapping pipeline (https://github.com/ArimaGenomics/mapping_pipeline/blob/master Arima_Mapping_UserGuide_A160156_v03.pdf), which is also recommended for data generated with the Arima library kit used in this work. I would like to ask the authors why this approach was not used.

 - It appears that the raw reads have been deposited in NCBI but are not yet publicly available.

 - In Figure 4, I am not sure why the red line corresponding to the hap1-only dataset is not shown in the graph. Is the shared green line fully covering the red one?

Are sufficient details of methods and materials provided to allow replication by others?

Yes

Is the rationale for creating the dataset(s) clearly described?

Yes

Are the datasets clearly presented in a useable and accessible format?

No

Are the protocols appropriate and is the work technically sound?

Yes

Reviewer Expertise:

genomics

I confirm that I have read this submission and believe that I have an appropriate level of expertise to confirm that it is of an acceptable scientific standard, however I have significant reservations, as outlined above.

# Response

**Published:** 2026-02-05

**Authors:** Tree of Life Team Sanger

**Affiliations:** Wellcome Trust Sanger Institute, UK

We thank the reviewer for their positive assessment of the genome assembly and for the constructive comments. **Genome annotation and RNA-seq data: **Genome annotation is performed by a third party, Ensembl at the European Bioinformatics Institute, and was not available when the first version of this data note was published. We now include a link to the annotation download page. **Figure 1: “live specimen”**: Figure 1 has been corrected **Use of tissue for different sequencing libraries: **The specimen was dissected on dry ice, with different tissues allocated to long-read, Hi-C and RNA sequencing. A standardised tissue strategy is followed within the Darwin Tree of Life programme, as described in Howard et al. (2025), which is already cited in the Methods. **Filtering of PacBio HiFi reads prior to *k*-mer analysis: **PacBio HiFi reads are subject to standard filtering during data generation before downstream analyses. **Hi-C read mapping strategy: **Hi-C reads were mapped using bwa-mem2, which is a faster implementation of bwa-mem and is also used within the Arima mapping pipeline. This approach was chosen to streamline processing and reduce runtime and disk usage. Reads were not filtered by mapping quality at this stage to retain information required for downstream quality control. **Availability of raw sequencing data**: The PacBio HiFi and Hi-C data can be accessed via the NCBI Sequence Read Archive at https://www.ncbi.nlm.nih.gov/sra/ERR13900449 and https://www.ncbi.nlm.nih.gov/sra/ERR13907227. **Figure 4: Merqury plot: **In Figure 4, the heterozygous peaks for haplotype 1 (red) and haplotype 2 (blue) are in the same place and are therefore not visible as separate curves.   The Tree of Life team, Wellcome Sanger Institute

# Reviewer report: Reviewer response for version 1

**DOI:** 10.21956/wellcomeopenres.27242.r129740

**Published:** 2025-09-04

**Authors:** Sina Beier

**Affiliations:** 1 Gurdon Institute, Cambridge, England, UK

The paper contains a detailed report on the genomic and transcriptomic study of the Poplar Admiral. it provides a high-quality genome paired with haplotype information and a mitochondrial genome. Additionally RNA-Seq data was generated, however the annotation using this data is unfortunately not part of this publication.

 Once the annotation is available, this will provide a valuable resource for a species that would lend itself to be an example in public engagement for the study of endangered species and adaptation as it is a known and visually pleasing butterfly that could pique interest.

Are sufficient details of methods and materials provided to allow replication by others?

Yes

Is the rationale for creating the dataset(s) clearly described?

Yes

Are the datasets clearly presented in a useable and accessible format?

Yes

Are the protocols appropriate and is the work technically sound?

Yes

Reviewer Expertise:

bioinformatics

I confirm that I have read this submission and believe that I have an appropriate level of expertise to confirm that it is of an acceptable scientific standard.
